# Effects of Exposure to a DNA Damaging Agent on the Hypoxia Inducible Factors in Organogenesis Stage Mouse Limbs

**DOI:** 10.1371/journal.pone.0051937

**Published:** 2012-12-14

**Authors:** Chunwei Huang, Barbara F. Hales

**Affiliations:** Department of Pharmacology and Therapeutics, McGill University, Montréal, Québec, Canada; Baylor College of Medicine, United States of America

## Abstract

Hypoxia plays a critical role in coordinating cell survival, differentiation and death in normal embryogenesis; during limb pattern formation, hypoxia affects two key processes, chondrogenesis and cell death. Hypoxia promotes chondrocyte differentiation and cartilage matrix synthesis and suppresses terminal differentiation. Depending on the context, hypoxia may induce cell cycle arrest, pro- or anti-apoptotic genes, or autophagy. The response to hypoxia is controlled by hypoxia inducible transcription factors, specifically *Hif1a*, *Hif2a* and *Hif3a*. Under normoxia, the hypoxia-inducible factors respond to a variety of stimuli that include several well established teratogens, such as retinoic acid, heavy metals and hyperglycemia. We hypothesize that teratogenic exposures disrupt limb development by altering the hypoxia signalling pathway. To test this hypothesis, we assessed the effects of a DNA damaging alkylating agent, 4-hydroperoxycyclophosphamide, on the hypoxia inducible factor (HIF) transcription factors and on hypoxia in the murine limb bud culture system. 4-Hydroperoxycyclophosphamide exposure increased HIF1 DNA binding activity and HIF1A and HIF2A, but not HIF3A, protein concentrations. HIF1A and HIF2A immunoreactivities were detected in the apical ectodermal ridge and interdigital regions, where cell death sculpts the limb; 4-hydroperoxycyclophosphamide treatment enhanced their immunoreactivities, specifically in these regions. In contrast, hypoxia was localized to areas of chondrogenesis, the cartilaginous anlagen of the developing long bone and phalanges, and was not enhanced by drug exposure. Thus, the exposure of limb buds *in vitro* to a DNA damaging teratogen triggered a hypoxia signalling response that was associated with cell death. During limb development the HIFs have oxygen-independent functions.

## Introduction

Early organogenesis stage mammalian embryos develop normally only in a relatively hypoxic environment. Indeed, exposure to O_2_ tensions that are either too low or too high has an adverse consequence. For example, early headfold stage rat embryos develop optimally in culture with 5% O_2_; exposure of gestation day 9 embryos to even physiological O_2_ (20%) results in neural tube malformations [1]. Hypoxia is important in the regulation of energy metabolism, heart development, angiogenesis, chondrogenesis, endochondral bone formation and cell death [2]. Hypoxia-inducible factors (HIFs) are transcription factors that play an essential role in the response of cells to hypoxia. The HIFA proteins form heterodimers with the HIFB subunit, previously identified as the aryl hydrocarbon receptor nuclear translocator (ARNT), and bind to the hypoxia response element in target genes that include phosphoglycerate kinase 1 (*Pgk1*), vascular endothelial growth factor (*Vegfa*), erythropoietin (*Epo*), and transforming growth factor β (*Tgfb1*), as well as pro-apoptotic genes, such as BCL2/adenovirus E1B interacting protein 3 (*Bnip3*) [3]. The O_2_-dependence of HIF transcription factor activity may be regulated at the level of transcription, translation, or post-translational modifications [3], but much of the literature has focused on protein stability. HIFA proteins are hydroxylated on specific amino acid residues by O_2_-dependent prolyl and asparaginyl hydroxylases; hydroxylated HIFa proteins are recognized and targeted for polyubiquitination and proteasomal degradation by von Hippel Lindau tumour suppressor protein (pVHL) [4]. Under hypoxic conditions, hydroxylation is reduced, binding to pVHL does not occur, and the HIFA proteins are stabilized, translocated to the nucleus, and interact with HIF1B [5]. Under conditions of normoxia, exogenous stimuli, such as retinoic acid, lipopolysaccharides, glucose, or maternal undernutrition, may alter HIF transcription factor activity but little is known about the underlying mechanisms [6], [7].

There are three *Hifa* genes, *Hif1a*, *Hif2a* and *Hif3a*. *Hif1a* and *Hif2a* are co-expressed in many cell types and have partially overlapping but non-redundant roles [8], [9]. The *Hif3a* mRNA is differentially spliced to produce multiple HIF3A isoforms that promote or inhibit the activities of HIF1A and HIF2A [10]. *Hif1a* expression is elevated in the cranial neuroepithelium, branchial arches, limb buds and tail of mid-gestation mouse embryos [11]. Mice that lack *Hif1a* have severe defects by gestation day 9.5, including open neural tube and heart malformations [12], [13]. Downregulation of *Hif1a*, associated with the administration of a retinoic acid receptor antagonist, produces cardiac defects [14], whereas overexpression is associated with an increased incidence of preeclampsia and intra-uterine growth retardation [15]. *Hif2a* (*Epas1* gene) is expressed predominantly in endothelial cells of the embryo and adult mouse [16]; *Hif2a* deficiency results in embryonic or perinatal mortality or survival with multiple organ deficiencies [17]. Homozygous mutant mice in which the first exon of *Hif3a* is replaced are viable but display enlargement of the right ventricle and impaired lung remodeling [18]. Together, these studies suggest that the HIFA proteins play critical, non-overlapping, roles in development.

**Figure 1:**
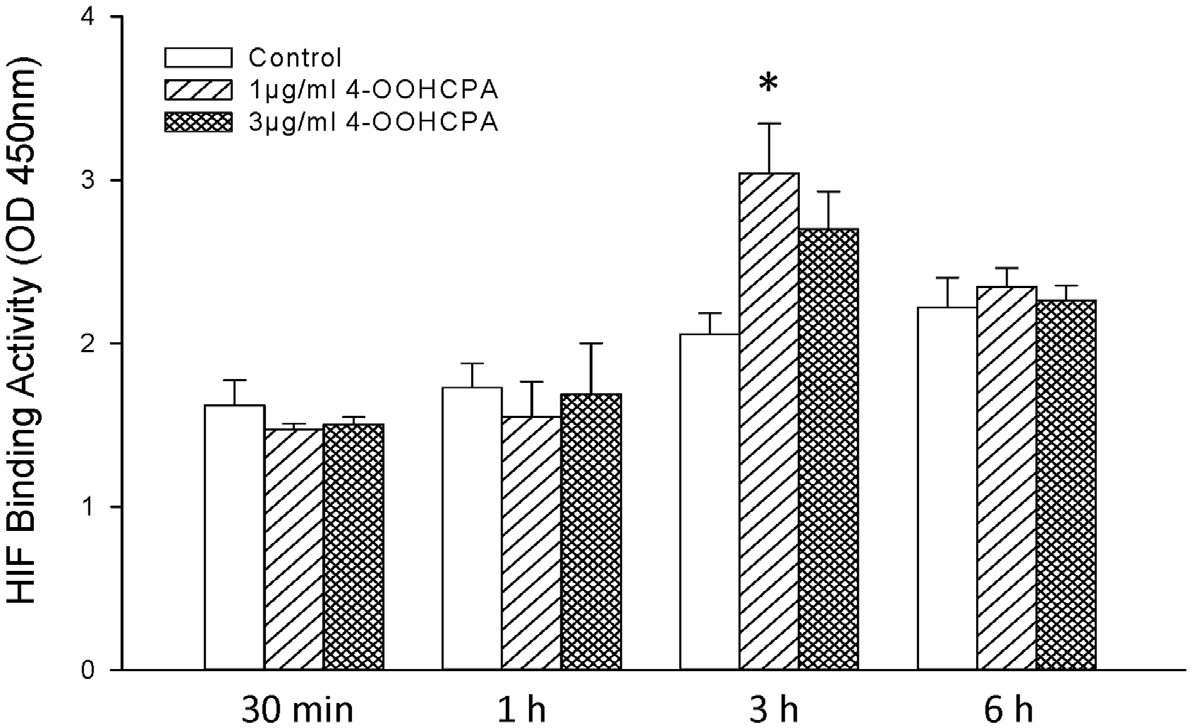
HIF1 heterodimer DNA binding activity in limbs exposed to 4-OOHCPA or vehicle. Limbs in culture were treated with saline (control) or 4-OOHCPA (1 µg/ml or 3 µg/ml) and collected after 30 min, 1, 3 or 6 h. HIF1 DNA binding activity was measured using an ELISA assay as described in the Methods. The data are expressed as mean ± SEM (µg nuclear extract standard/µg sample protein); each bar represents 3–4 litters. * indicates a significant difference from control at that time point (P<0.05).

**Table 1:** Effects of 4-OOHCPA exposure on the expression of genes regulated by HIF1A.

Treatment time	Gene Symbol	Fold change	t-test
3 h	*Igf2*	1.55	0.29
	*Mmp2*	1.85*	0.03
6 h	*Bcl2*	1.92	0.14
	*Hif2a*	1.58	0.28
	*Pdk1*	−1.52	0.08

* indicates a significant difference from control.

Chondrogenesis and apoptosis are key processes in limb development [19]. Mesenchymal cells form pre-chondrogenic condensations and differentiate into the cartilage anlagen of the limb, taking on their regional identities in a temporal proximal-distal fashion. Programmed cell death is the basic mechanism by which the limb is shaped. There is evidence that the HIF proteins play an important role in the regulation of chondrogenesis and cell death. HIF1A and HIF2A activate expression of *Sox9,* a key regulator of chondrogenesis [20], [21]. Deletion of *Hif1a* early or late during chondrogenesis leads to cell death in the proximal limb bones and severe limb shortening [22], [23].

**Figure 2:**
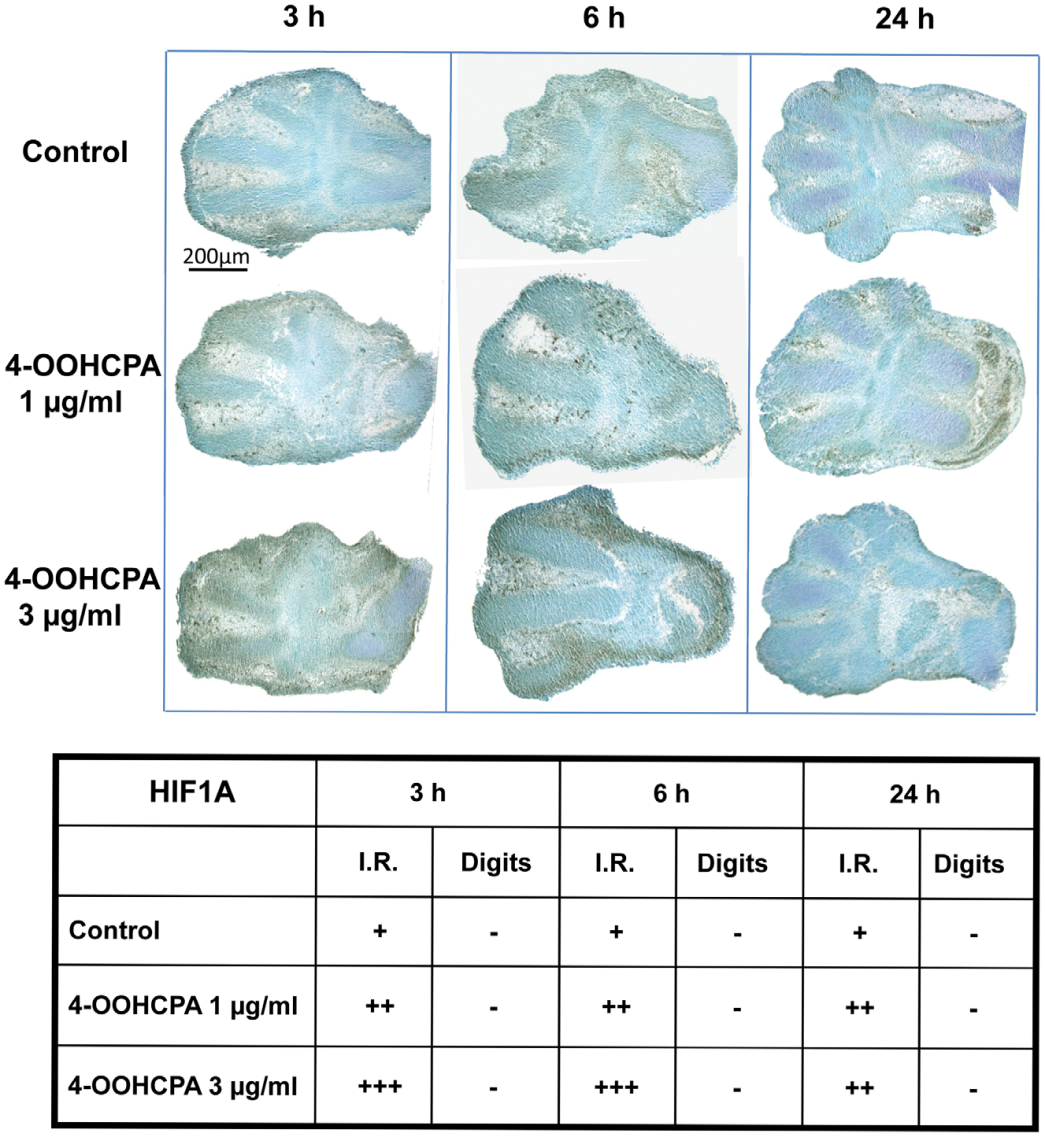
Immunolocalization of HIF1A in limbs. Immunoreactivity was detected in control limbs in the apical ectodermal ridge and interdigital regions (I.R.). The mesenchymal condensations/developing cartilaginous anlagen (Digits) were not immunoreactive. 4-OOHCPA treatment resulted in a concentration- and time-dependent increase in HIF1A immunoreactivity in this area in the 3 and 6 h exposure groups; staining was diminished by 24 h. Four separate replicates were done.

In animal studies, temporary periods of hypoxia, due to a constriction of the uterine arteries or exposure to drugs that induce hypoxia by affecting the maternal cardiovascular system, lead to birth defects such as transverse limb reduction defects or heart defects [24]. The culture of gestation day 10.5 rat embryos in 10–15% O_2_ increased the incidence of malformations and the extent of oxidative stress [25]. Our interest in the role of hypoxia signalling in mediating the effects of teratogenic exposures on embryo development was triggered by the observation that exposure to a DNA damaging teratogen, 4-hydroperoxycyclophosphamide (4-OOHCPA), increased *Hif1a* expression in cultured murine limb buds [26]. The teratogenicity of cyclophosphamide, an anticancer nitrogen mustard drug, has been well characterized in several species in vivo [27]. 4-OOHCPA, a preactivated analog of cyclophosphamide, causes growth reduction and digit defects in the limb bud culture system [28]; since this is an *in vitro* model, the effect is direct, i.e. not due to the induction of hypoxia in the dam.

**Figure 3:**
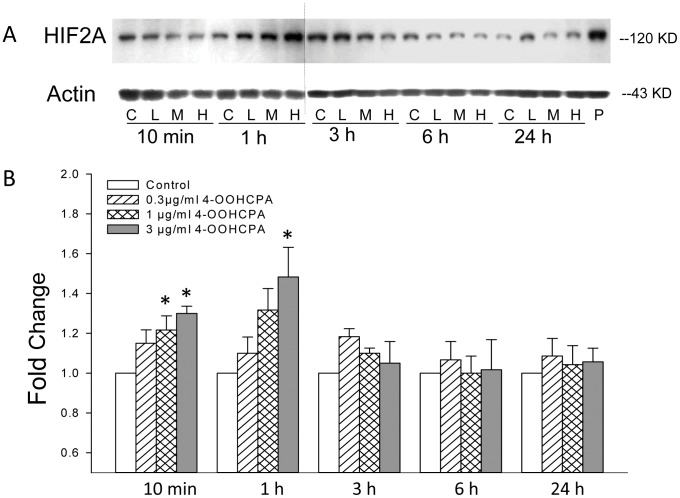
4-OOHCPA exposure induced HIF2A. A: Western blot analysis of HIF2A protein (118KD) and actin (43KD) in limbs at 10 min, 1, 3, 6, and 24 h after treatment with 4-OOHCPA at 0.3 µg/ml (L) 1.0 µg/ml (M) or 3.0 µg/ml (H). P represents the positive control. B: Fold changes from the scan densitometry quantification of the HIF2A band in drug treated groups compared to control groups. Hatched bar: 0.3 µg/ml (L), cross-hatched bar: 1.0 µg/ml (M) and gray bar: 3.0 µg/ml (H). Each bar (mean ± SEM) represents six to seven replicates; bars with an asterisk are significantly different from control at the same time point (p ≤0.05).

We hypothesize that teratogenic exposures disrupt limb development by altering the hypoxia signalling pathway. To test this hypothesis, we determined the impact of exposure to 4-OOHCPA on HIF DNA binding activity and the expression and activity of the HIF transcription factors in limbs. Furthermore, to elucidate the oxygen-dependence of this HIF response, we compared the localization of HIFA proteins with that of hypoxia in limb. Together, the results of this study reveal that the HIFs have oxygen-independent functions during limb development, particularly in regulating cell death.

**Figure 4:**
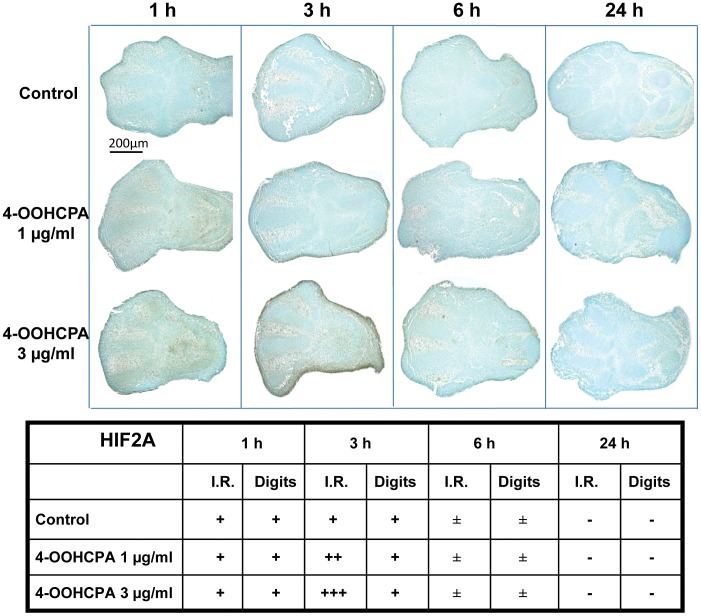
Localization of HIF2A immunoreactivity in limbs. HIF-2A reactivity was detected in control limbs at 1 and 3 h in the apical ectodermal ridge, interdigital area (I.R.) and developing cartilaginous anlagen (Digits). 4-OOHCPA exposure increased HIF2A immunoreactivity in the apical ectodermal ridge and interdigital regions (I.R.) at 3 h. No differences were observed between control and drug-treated limbs after 6 h or 24 h of culture. Four separate replicates were done.

## Materials and Methods

### Limb Bud Cultures and Drug Treatments

#### Ethics statement

This study was done in accordance with the guidelines of the Canadian Council on Animal Care for the ethical use and care of animals in science. The protocol (Protocol Number 1825) was approved by the Animal Care Committee of McGill University.

**Figure 5:**
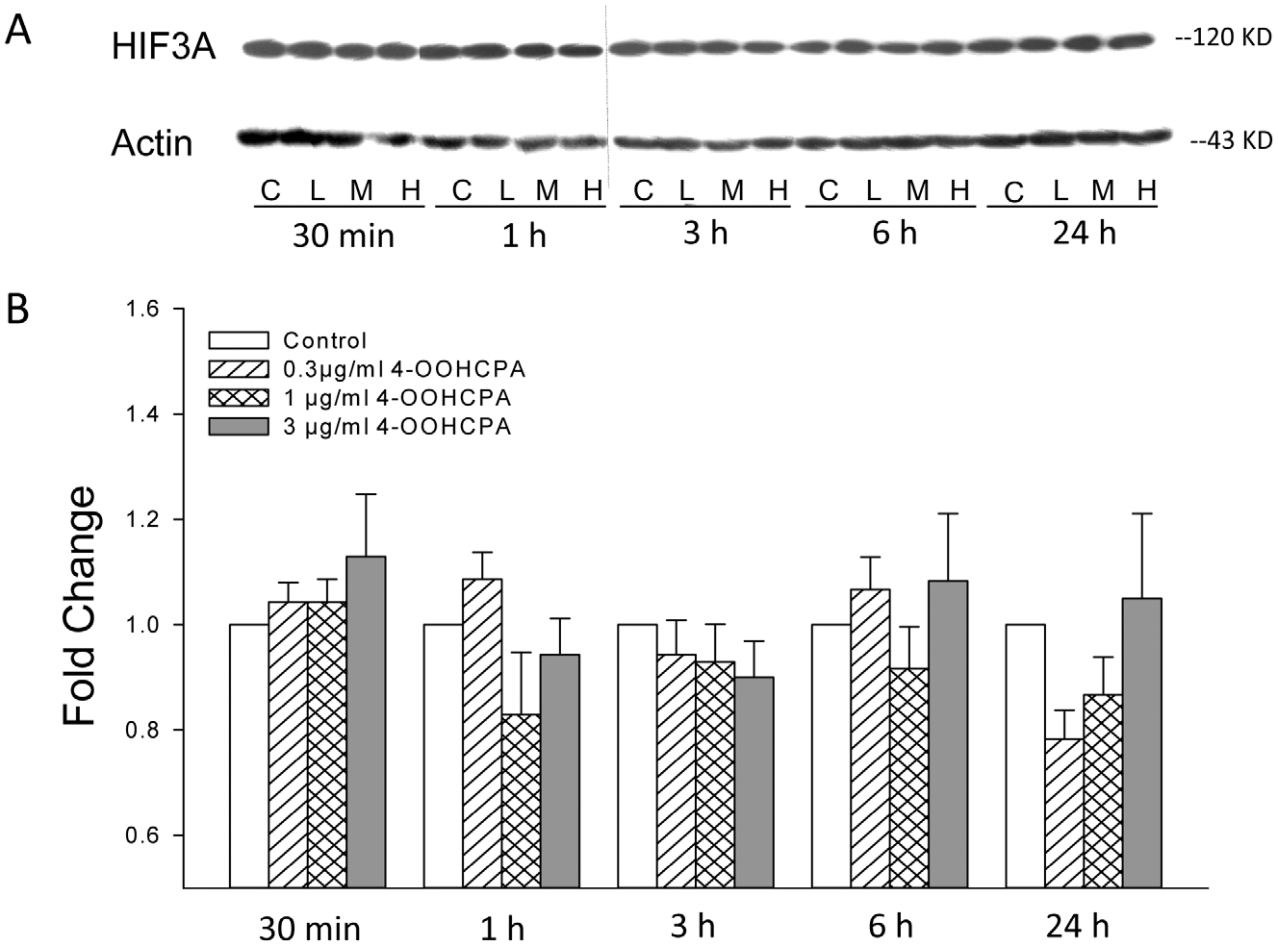
Western blot of HIF3A. HIF3A protein was detected in control limbs at all time points. A: Western blot analysis of HIF3A protein (118 KD) and actin (43 KD) in limbs at 30 min, 1, 3, 6, and 24 h after treatment with 4-OOHCPA at 0.3 µg/ml (L) 1.0 µg/ml (M) or 3.0 µg/ml (H). B: Densitometric quantification of HIF3A in drug treated groups compared to control groups. Hatched bar: 0.3 µg/ml (L), cross-hatched bar: 1.0 µg/ml (M) and gray bar: 3.0 µg/ml (H). HIF3A protein concentrations were not affected by drug treatment or time. Each bar (mean ± SEM) represents six to seven replicates.

Timed-pregnant CD1 mice (20–25 g), mated between 8∶00 and 10∶00 (gestation day 0), were purchased from Charles River Canada Inc. (St. Constant, QC) and housed at the McIntyre Animal Centre (McGill University, Montréal, QC). Between 8∶00 and 10∶00 on gestation day 12, females were killed by CO_2_ overdose and cervical dislocation, uteri were removed, and the embryos were dissected out in sterile Hank’s Balanced Salt Solution; the embryos at this time were 5–6 mm in length with about 42 somite pairs. Forelimbs were excised just lateral to the somites, pooled, and cultured in 6 ml of culture medium consisting of 75% BGJb Medium (GIBCO BRL Products, Burlington, ON) and 25% salt solution supplemented with ascorbic acid (160 µg/ml) and gentamycin (1 µl/ml, GIBCO BRL Products), as previously described [26], [28]. 4-Hydroperoxycyclophosphamide (4-OOHCPA; a gift from Dr. S. Ludeman) or distilled water was added to the designated cultures. The cultures were terminated at the specified times.

**Figure 6:**
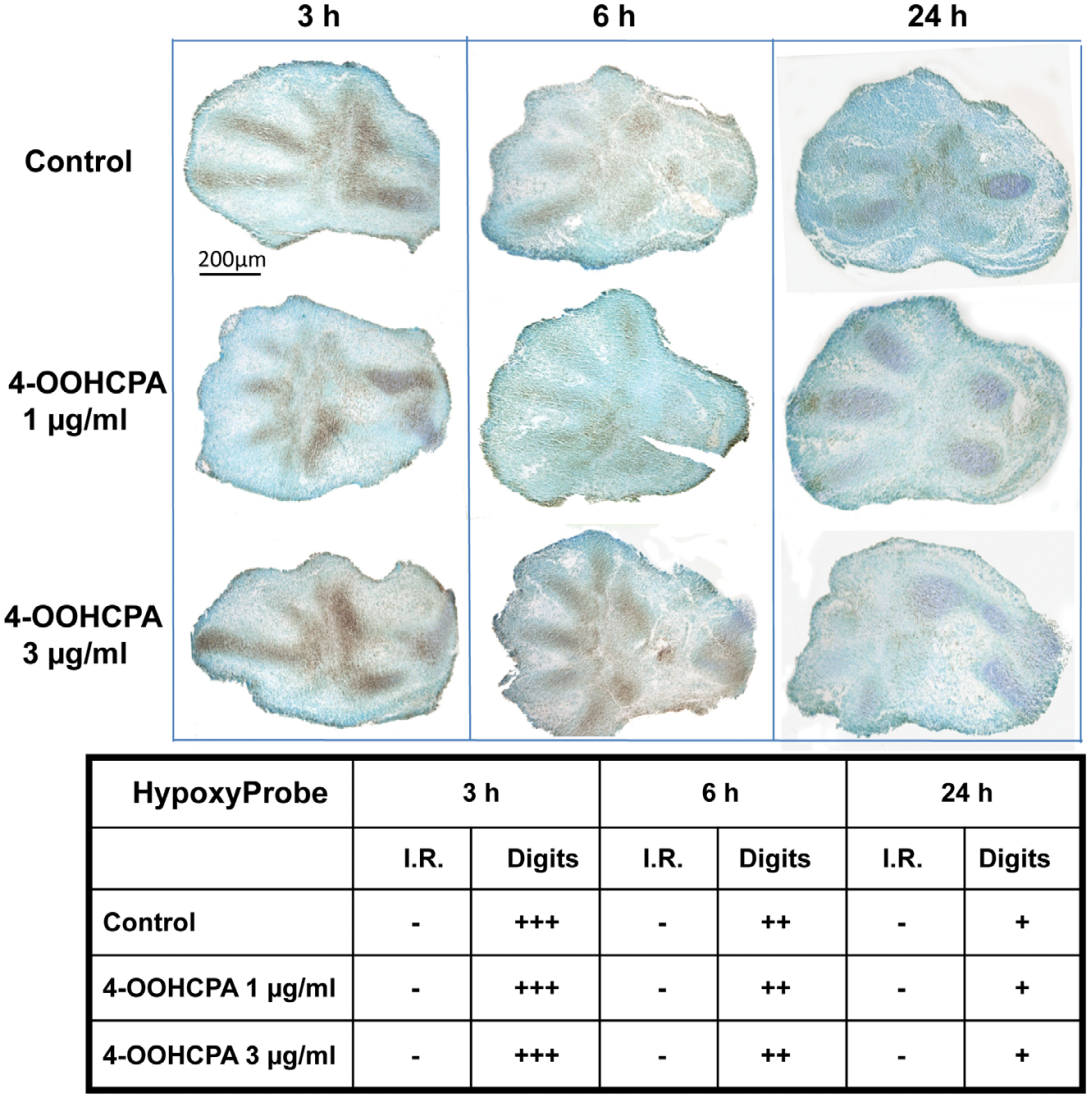
Detection of hypoxia in limbs using Hypoxyprobe. Hypoxyprobe™-1 reactivity was detected in the digit and core areas (Digit) of limbs where chondrogenesis occurs; staining in these areas was strong at 3 h and 6 h and diminished at 24 h. Hypoxyprobe reactivity was not observed in the interdigital regions (I.R.). No differences were observed between control and drug-treated limbs. Four separate replicates were done.

### HIF1 DNA Binding Activity Determination with ELISA

Limbs were cultured for 0.5, 1, 3, or 6 h, in the presence or absence of 4-OOHCPA (1 µg/ml or 3 µg/ml). In previous studies, we showed that exposure to these concentrations of 4-OOHCPA had marked effects on long bone outgrowth, digit formation and cell death; HIF1A expression was induced by 3 h [26], [28]. Eight fresh limbs from each treatment group were used to prepare nuclear extracts (Nuclear Extract Kit, Active Motif, Carlsbad, CA) following the manufacturer's recommendations. Nuclear protein concentrations were assessed with the Bio-Rad Laboratories protein spectrophotometric assay (Mississauga, ON, Canada). The HIF1 heterodimer complex DNA binding activity was detected using TransAM™ Chemi Kits (Active Motif) in which an oligonucleotide containing the hypoxia response element from the *Epo* gene (5′- TACGTGCT-3′) is immobilized on 96 well plates. The primary antibody recognizes an epitope on HIF1A that is accessible after DNA binding; conjugation of the secondary antibody to horseradish peroxidase provides a colorimetric readout. Three or four separate replicates (each with limbs obtained from different litters) were done for each treatment and time point; values represent means ± SEM. The data were analyzed by one-way ANOVA using the SigmaPlot version 12 software program (Systat Software Inc., Chicago, IL). The level of significance was taken as P<0.05.

**Figure 7:**
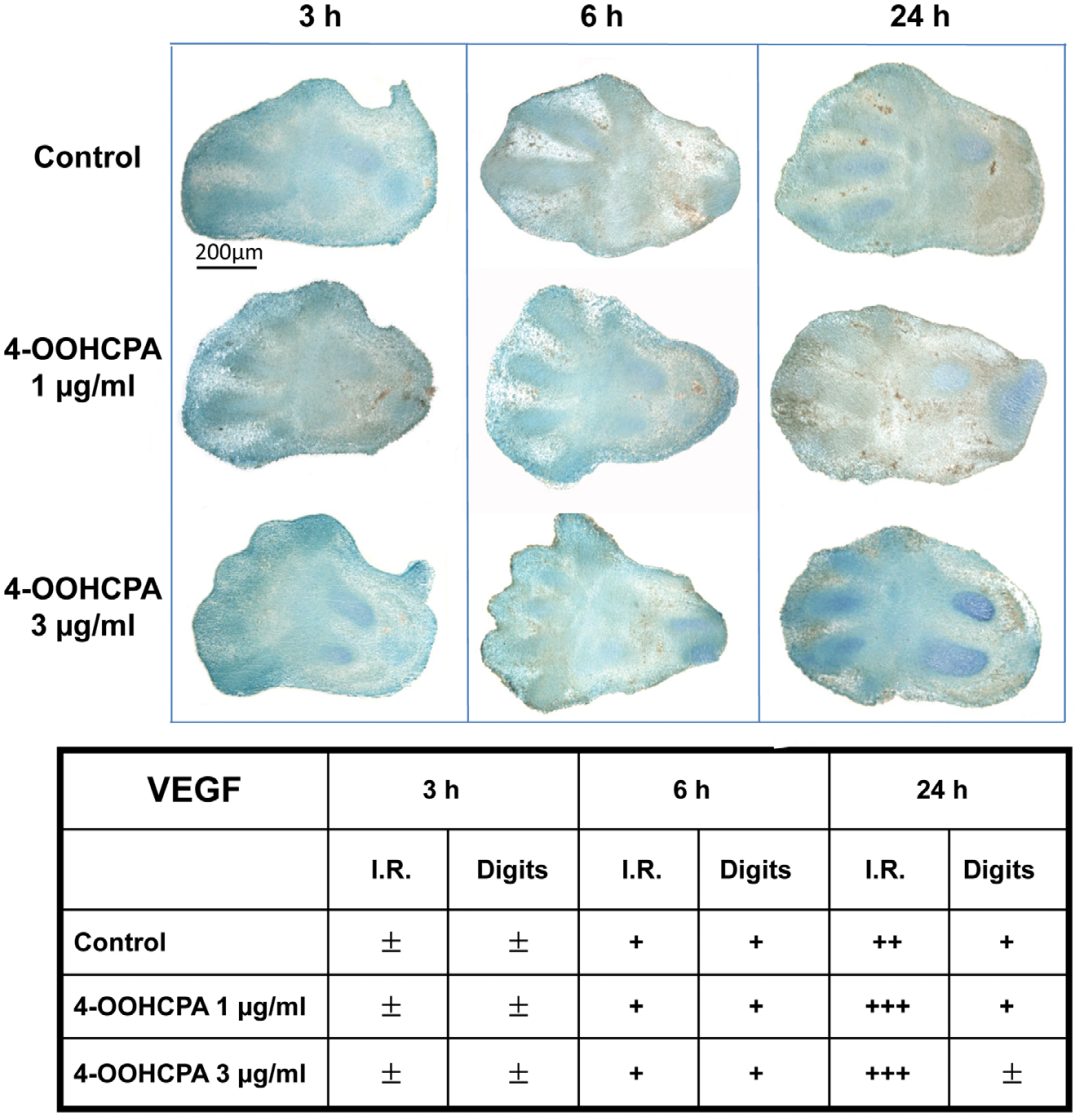
Immunolocalization of VEGF in control and 4-OOHCPA treated limbs. VEGF protein was detected in control limbs in the apical ectodermal ridge and interdigital regions (I.R.) with stronger staining observed at 24 h. Staining was increased in these areas in limbs exposed to either 1.0 or 3.0 µg/ml 4-HOOCPA for 24 h. Four separate replicates were done.

### Mouse HIF-Regulated cDNA Plate Arrays

Consequences on target gene expression were assessed in limbs cultured in the presence or absence of 1 µg/ml of 4-OOHCPA for 3 or 6 h (since the HIF1 DNA binding activity experiments, described above, revealed that binding was increased significantly in limbs exposed to this concentration for 3 h). Total RNA was isolated using RNeasy® Micro Kits (Qiagen, Mississauga, ON), following the manufacturer’s recommendations. The RNA (2.5 µg/sample) was reverse transcribed into cDNA in the presence of biotin-dUTP and then hybridized to Mouse HIF-Regulated cDNA Plate Arrays pre-coated with the specific oligonucleotides for 23 HIF-regulated genes (Signosis, Inc., Sunnyvale, CA). The cDNAs bound to these oligonucleotides were detected with streptavidin-horseradish peroxidase using a horseradish peroxidase chemiluminescent substrate and the LUMIstar Galaxy plate reader (BMG Labtech/Fisher Scientific, Nepean, ON, Canada). Procedures for the generation of the biotin-labeled cDNAs by *in vitro* transcription, the hybridization to the array, washing, and scanning were all conducted in accordance with the manufacturer’s recommendations. Five separate replicates were done for each treatment and time point.

The raw chemiluminescent intensities obtained from the LUMIstar Galaxy plate reader were imported into GeneSpring (version 11.5.1; Agilent Technologies, Palo Alto, CA) for analysis. To minimize experimental variation, the ratio of the cDNA intensity for the 18S rRNA of each replicate to the average 18S intensity for all replicates, including both control and treated groups, was used as an internal control. Thus, the intensity of each gene was adjusted for the 18S ratio of the replicate. Changes in gene expression were considered only when the difference in expression level was at least 1.5-fold control; a 1.5-fold change is equivalent to an increase by 50% or a decrease by 33%. The expression of genes in the 4-OOHCPA treated limbs that passed this ≥1.5-fold change filter was compared to that in control limbs by the Student’s *t*-test with the Benjamini and Hochberg multiple comparisons correction and a false detection rate of 5%.

### Real-time qRT-PCR Analysis of Matrix Metallopeptidase 2 (*Mmp2*) Gene Expression

Limbs were cultured in the presence or absence of 4-OOHCPA (1 µg/ml) for 3 or 6 h and then stored in RNAlater RNA Stabilization Reagent (Qiagen) at −20°C. Total RNA for real-time qRT-PCR analysis was isolated using the RNeasy® Micro Kit (Qiagen), following the manufacturer’s recommendations. qRT-PCR analysis of *Mmp2* gene expression was done using One step QuantiTect® SYBR® Green RT-PCR analysis (Qiagen). First-strand cDNA was synthesized from 10 ng of total RNA using an optimized blend of Omniscript and Sensiscript Reverse Transcriptases (Qiagen). The primers were generated with Primer3 software (http://frodo.wi.mit.edu) and produced by Alpha DNA (Montreal, QC, Canada). Reactions containing 10 pmol each of the left (TTCAACGGTCGGGAATACA) and right (AGCCATACTTGCCATCCTTC) primers and the SYBR Green PT-PCR Mix were amplified in a final volume of 20 µl. Cycling parameters were: 50°C for 20 min for the reverse transcription reaction, 95°C for 15 min for primer activation, followed by 45 cycles of denaturation at 94°C for 15 s, annealing at 55°C for 30 s, and primer extension at 72°C for 20 s. Amplification reactions and fluorescent measurements were done using a real-time LightCycler® (Roche Diagnostics, Laval, QC, Canada). A melting curve analysis was done on all samples to confirm the amplification specificity. The relative quantification data were obtained by comparison of the sample fluorescence with a standard curve that was amplified in parallel; this standard curve consisted of first-strand cDNA generated from hindlimb total RNA. Parallel amplification of 18S rRNA (*Rn18s*) was used as an internal control to normalize the gene expression (left primer: AAACGGCTACCACATCCAAG, right primer: CCTCCAATGGATCCTCGTTA). Amplifications were done in duplicate; the results are from six independent cultures of each treatment group, with a minimum of four limbs per replicate. Data were analyzed using one-way ANOVA and the post-hoc Dunnett multiple comparison test (SigmaPlot 12, Systat Software, Inc., San Jose, CA). The level of significance was taken as *p*≤0.05.

### Western Blot Analysis

At the designated times, four to eight limbs per group were placed in liquid N_2_ and stored at −80°C. Lysates were prepared by sonicating limbs with high intensity ultrasonic liquid processors (Sonics & Materials Inc, Danbury, CT) for 2–3 seconds in lysis buffer (50 mM Tris, pH 7.5, 150 mM NaCl, 0.1% SDS, 0.5% sodium deoxycholate, 0.2 M phenylmethylsulfonyl fluoride, 10 µg/ml leupeptin, 3 µg/ml aprotinin, 40 µg/ml bestatin, and 1% Nonidet P-40). The lysates were cleared by centrifugation for 10 min at 10,000 rpm at 4°C and denatured at 85°C for 5 min. Protein concentrations were assessed with the Bio-Rad Laboratories protein spectrophotometric assay (Mississauga, ON, Canada). Samples (25 µg protein) were fractionated by SDS-PAGE using 4–20% Novex® Tris-Glycine gels (Life Technologies Inc., Burlington, ON, Canada). Novex® Sharp Standards (Life Technologies Inc.) were used as molecular weight markers. Lysates of limbs cultures treated with deferoxamine mesylate salt (1 mM, 6 h, Sigma-Aldrich, St-Louis, MO), an iron chelator that acts as an hypoxia-mimetic agent, and hypoxia-induced COS-7 nuclear extracts (Novus Biologicals, Inc., Oakville, ON, Canada) were used as positive controls.

The fractionated proteins were transferred to a PVDF membrane using iBlot® Western Detection Kits (Life Technologies Inc.). The blots were blocked with 10% nonfat dried milk, in TBS-T (137 mM NaC1, 20 mM Tris [pH 7.4], 0.1% Tween® 20) at room temperature for 1 h and then incubated with the antibody to HIF2A (Novus Biologicals, Inc., primary mouse monoclonal, NB100–132, 1∶1,000), or HIF3A (Abcam, primary rabbit polyclonal antibody to ab2165, 1∶1,000) in 5% nonfat dried milk, in TBS-T overnight at 4°C. The secondary antibodies, sheep anti-mouse IgG (1∶2,000, GE Healthcare Limited, Baie d’Urfé, QC, Canada) or donkey anti-rabbit antibody (1∶10,000, GE Healthcare Limited) conjugated to horseradish peroxidase, were used to detect specific antibody interactions.

Western blots were visualized with the Enhanced Chemiluminescence Plus Kit (GE Healthcare Limited) and CL-XPosure film (Fisher Scientific). Quantification of western blot data was done using a ChemiImager 4000 imaging system (Alpha Innotech, San Leandro, CA) with AlphaEase 3.3b software. Actin (1∶5,000) protein expression was monitored as a loading control, using a donkey anti-goat polyclonal antibody (1∶10,000, [0.04 µg/ml] Santa Cruz Biotechnology, Santa Cruz, CA). Six or seven separate replicates were done for each experiment; values represent means ± SEM. The data were analyzed by two-way ANOVA using the SigmaPlot version 12 software program (Systat Software Inc., Chicago, IL). The level of significance was taken as P≤0.05.

### Immunohistochemical Localization of HIF1A, HIF2A, and VEGF

Limbs were cultured for 3, 6 or 24 h, fixed with 4% paraformaldehyde at 4°C for 4 h, dehydrated with ascending ethanol concentrations, cleared with xylene, and embedded in paraffin. Sections (5 µm) were cut, mounted on slides, deparaffinized with xylene, and rehydrated with descending concentrations of ethanol. To detect HIF1A an antigen unmasking step was necessary; these sections were heated in a microwave oven for a total of 15 min (2 min with high power and 13 min with 10% power, cooled at room temperature for 15 min) in 0.1 M sodium citrate. Following this, the sections were washed with TBS (137 mM NaC1, 20 mM Tris, pH 7.4), treated with 0.45% H_2_O_2_ in methanol for 25 min, washed with TBS, blocked with 1.5% normal horse serum in TBS for 30 min, and then incubated with rabbit polyclonal antibodies to HIF1A (Novus Biologicals, Inc., NB100–449, at 1∶50 dilution, for 1 h at room temperature) or VEGF (Santa Cruz, SC-507, at a dilution of 1∶100, overnight at 4°C). After washing with TBS, sections were stained via the avidin biotin-peroxidase complex method with a Vectastain Elite ABC kit (Vector Laboratories Inc., Burlingame, CA). Each section was reacted with 3,3' diaminobenzidine (DAB) substrate to detect peroxidase using an ImmPACT™ DAB substrate kit (Vector Laboratories Inc), and then counterstained with methylene blue. Four separate replicates were done for each experiment.

The preparation of sections for the analysis of HIF2A immunohistochemistry was similar except for the following modifications. Sections were cleared and deparaffinized with Histo-Clear (National Diagnostics/Diamed Lab Supplies, Mississauga, ON, Canada). They were washed with PBS, treated with 0.3% H_2_O_2_ in water for 5 min, washed with PBS, blocked with M.O.M. reagent (mouse-on-mouse, Vector Laboratories Inc.) in PBS for 1 h, and then incubated with the mouse monoclonal antibody for HIF2A (Novus Biologicals, Inc., NB100–132, at 1∶40 dilution for 30 min) at room temperature.

### Detection of Hypoxia Using Hypoxyprobe

Timed pregnant CD1 gestation day 12 mice were injected with pimonidazole, a marker of tissue hypoxia (60 mg/kg i.v.; NPI-Hypoxyprobe Inc., Burlington, MA) [29]. The mice were euthanized 1 h later; forelimbs were excised and cultured in the absence or presence of 4-OOHCPA (1.0 or 3.0 µg/ml) for 3, 6, or 24 h. Limbs were fixed with 4% paraformaldehyde at 4°C for 4 h, dehydrated with ascending ethanol concentrations, cleared with xylene, and embedded in paraffin. Sections (5 µm) were cut, mounted on slides, deparaffinized with xylene, and rehydrated with descending concentrations of ethanol. The sections were washed with TBS, treated with 3.0% H_2_O_2_ in water for 5 min, washed with TBS, blocked with IHC Select blocking Reagent (EMD Millipore, Billerica, MA) for 5 min, and then incubated with a conjugate between the Hypoxyprobe™-1 monoclonal antibody and fluorescein isothiocyanate (FITC) (NPI-Hypoxyprobe Inc.) at a 1∶200 dilution for 30 min at room temperature. After washing with TBS, sections were incubated with a secondary conjugate for 30 min. Each section was reacted with DAB substrate for peroxidase using an ImmPACT™ DAB substrate kit (Vector Laboratories Inc), and counterstained with methylene blue, as described above. Four separate replicates were done for this experiment.

## Results

### Effects of 4-OOHCPA Treatment on HIF1 DNA Binding Activity and on the Expression of HIF1-Responsive Genes

The relative DNA binding activity of HIF1 heterodimers was transiently enhanced by exposure of limbs to 4-OOHCPA for 3 h (Fig. 1). To determine the impact of this increase in DNA binding activity on gene expression, we profiled the expression of 23 HIF1 dependent genes in control and 4-OOHCPA (1 µg/ml) exposed limbs using cDNA plate arrays (Table 1, Supplementary data, Table S1). Four genes, *Crebp*, *Glut1*, *Vhl*, and *Ep300*, were highly expressed (≥2 fold the average expression of the array, Supplementary data, Table S1) in both the control and drug treated groups. Treatment with 4-OOHCPA for 3 h upregulated the expression of *Igf2* and *Mmp2* (≥1.5 fold); exposure for 6 h upregulated the expression of *Bcl2* and *Hif2a* and downregulated the expression of *Pdk1* (Table 1). qRT-PCR analysis of *Mmp2* expression confirmed that this gene was upregulated in limbs exposed to 4-OOHCPA at 3 h (1.26±0.08, mean ± SEM, n = 6; p≤0.05); by 6 h, *Mmp2* expression did not differ between the drug-treated and control limbs (0.93±0.09, n = 6).

### Localization of HIF1A Immunoreactivity in Control and 4-OOHCPA Treated Limbs

In a previous study, we reported that exposure to 4-OOHCPA for 6 h resulted in a concentration-dependent increase in HIF1A protein concentration (0.3 µg/ml: 1.78 fold; 1.0 µg/ml: 1.84 fold; 3.0 µg/ml: 2.36 fold) compared to vehicle treated controls [26]; this induction was not observed at 3 h and did not persist to 24 h. In this study, we determined the localization of this HIF1A immunoreactivity in control and 4-OOHCPA treated limbs (Fig. 2). In control limbs, immunoreactivity was detected in isolated cells in the apical ectodermal ridge and interdigital regions (denoted as I.R.). A comparison of the limbs cultured for 3, 6 or 24 h revealed that time in culture did not have an appreciable effect on HIF1A staining. However, treatment with 4-OOHCPA produced a dramatic increase in HIF1A immunoreactivity in the 3 and 6 h exposure groups; by 24 h staining was diminished. This increase in HIF1A immunoreactivity in the presence of 4-OOHCPA was also largely localized to the apical ectodermal ridge and the interdigital regions.

### Effects of 4-OOHCPA Exposure on HIF2A and HIF3A Protein Expression

Since the three *Hif1a* genes have complimentary functions, we were interested in the effects of 4-OOHCPA exposure on the expression of HIF2A and HIF3A in limbs. Although *Hif2a* mRNA concentrations in limbs exposed to 4-OOHCPA for 6 h were increased (1.58 fold, relative to control), this increase was not statistically significant (Table 1). However, Western blot analysis revealed that 4-OOHCPA exposure increased the protein concentrations of HIF2A (Fig. 3). This induction was both concentration- and time-dependent. Interestingly, increased HIF2A was observed as early as 10 minutes after 4-OOHCPA exposure and peaked at 1 h; limbs cultured for 3, 6 or 24 h did not differ from controls.

HIF2A immunoreactivity was detected in control limbs cultured for 1 or 3 h in the apical ectodermal ridge, interdigital regions and the core mesenchymal condensations (Fig. 4). 4-OOHCPA exposure for 3 h increased HIF2A immunoreactivity in the interdigital regions and apical ectodermal ridge (I.R.) areas. No marked differences in HIF2A immunoreactivity were observed between the control and drug-treated limbs after 1, 6 or 24 h of culture.

HIF3A protein was detected in control limbs at all time points tested (Fig. 5). Since drug treatment did not alter HIF3A protein concentrations in limbs we did not assess the localization of this protein.

### Localization of Hypoxia in Limbs

Pimonidazole immunoreactivity (Hypoxyprobe) was used as a marker to determine the localization of hypoxia in cultured limb buds (Fig. 6). Hypoxia was detected in control limbs in the regions undergoing chondrogenesis, i.e. the forming cartilaginous anlagen of the long bones and digits (denoted as “Digits”). Maximal reactivity was detected in the control limbs cultured for 3 h; the signal decreased by 6 h and was faint after culture for 24 h. Limbs exposed to 4-OOHCPA did not differ noticeably from controls, with immunoreactivity remaining focused in the mesenchymal condensations and no change in the intensity of staining.

### Immunolocalization of VEGF in 4-OOHCPA-treated and Control Limbs

*Vegfa* expression is regulated by both HIF1A and HIF2A [8]; previously, we reported that the exposure of limbs to 4-OOHCPA upregulated *Vegfa* expression [26]. In this study, there was an apparent increase in *Vegfa* expression (1.45 fold increase in limbs exposed to 1 µg/ml of 4-OOHCPA for 3 h) but this increase did not reach our >1.5 fold cut-off value (Supplementary data, Table S1). VEGFA immunoreactivity in limbs is shown in Figure 7. VEGF immunoreactivity was detected in control limbs in the apical ectodermal ridge, interdigital and mesenchymal condensations; staining was stronger at 24 h than at 3 or 6 h. Exposure to 4-OOHCPA did not visibly enhance VEGF immunoreactivity in the cartilaginous anlagen or digits; after 24 h an increase in staining in the interdigital areas was observed.

## Discussion

Here, we demonstrate that the exposure of developing limb buds to 4-OOHCPA induces HIF1A and HIF2A, but not HIF3A. HIF1A and HIF2A immunoreactivities are enhanced in the apical ectodermal ridge and interdigital regions, where programmed cell death occurs. In contrast, hypoxia is localized to the cartilaginous anlagen formed by the condensation of mesenchymal cells and is not enhanced by drug exposure. VEGF, an HIF target gene, immunoreactivity was found in both the apical ectodermal ridge/interdigital area and in the mesenchymal condensations but was not visibly affected by 4-OOHCPA exposure. Thus, exposure to this DNA damaging agent induces the hypoxia response signalling pathway in limb buds in the absence of an effect on hypoxia.

Under normoxia, the stimuli that have been reported to induce HIF1 transcription factor activity include growth factors, insulin, and glucose [30]. Cyclophosphamide, a bifunctional nitrogen mustard, alkylates DNA to form crosslinks, resulting in single and double strand breaks. The mechanism by which such a DNA damaging agent might induce HIF proteins in the absence of hypoxia is not known. One possibility is that 4-OOHCPA-induced DNA damage activates Sirt1, a class III histone deacetylase. Previous studies have shown that Sirt1 activation increases HIF2A signalling and *Epo* gene expression [31].

Exposure to 4-OOHCPA increased the steady state concentrations of both the mRNA and protein concentrations of *Hif1a and Hif2a* in limbs, without influencing HIF3A protein concentrations. Thus, the expression of HIFA family members in limbs is differentially regulated and may occur at multiple levels. Based on the time course of induction, we speculate that DNA damage may regulate HIF1A transcription and translation. The rapidity of the increase in HIF2A protein concentrations (10 min) suggests that the primary effect may be on protein stability, probably mediated via inhibition of proteasomal degradation; the induction of *Hif2a* mRNA concentrations is a later response, not observed until 6 h after drug exposure. In the context of these data, it is interesting that the exposure of human bronchial epithelial cells to inorganic arsenic increased the expression of HIF2A, but not HIF1A, by inhibiting its degradation through the ubiquitin-mediated proteasome pathway [32].

Hypoxia plays a major role in limb development by activating the expression *Sox9,* a key regulator of chondrogenesis [21], [23], and by inhibiting expression of *Runx2*, affecting osteogenesis [33]. We observed hypoxia in the cartilaginous anlagen of cultured limbs in the absence of appreciable HIF1A immunoreactivity. Conditional knockout experiments provide data suggesting that the HIF proteins are not required for early events during chondrogenesis. Indeed, conditional inactivation of *Hif1a* did not affect either the formation of precartilaginous condensations or *Sox9* expression in gestation day 12.5 mouse embryos, although there was a delay in cartilage formation by gestation day 13.5 [33], [34]. Conditional inactivation of *Hif2a* in limbs revealed that *Hif2a* is also not required for the differentiation of mesenchymal cells into chondrocytes; here, no limb phenotype was observed until gestation day 15.5 [35].

The apical ectodermal ridge and interdigital regions of limbs exposed 4-OOHCPA display increased apoptotic cell death; pretreatment with an inhibitor of caspase 3 activation, DEVD-CHO, provided partial protection in 4-OOHCPA exposed limbs [36]. The p53 tumor suppressor gene signaling pathway plays a protective role in regulating apoptosis in the limbs; interestingly, a p53-dependent switch in the type of cell death, from apoptosis to necrosis, was observed in limbs exposed to 4-OOHCPA [37]. A number of studies have linked hypoxia, increased HIF1A, and increased p53 to an increase in apoptosis. An et al. [38] reported that HIF1a overexpression under normoxia increased p53 activity. Here again, HIF1a and HIF2a may have opposite roles. However, since DNA damage itself activates p53, it is not possible to attribute a downstream increase in p53-dependent apoptosis to the observed increase in HIFA immunoreactivity in the apical ectodermal ridge and interdigital regions. It is clear that the interactions between HIFs and p53 are complex [39].

Thus, hypoxia signalling pathways are activated in limbs exposed to a developmental toxicant in a region and time-specific manner. The oxygen-independent functions of this signalling pathway may play a role in mediating the response to a variety of exogenous exposures.

## Supporting Information

Table S1 HIF cDNA plate array data.
(XLSX)

## References

[1] Morris GM, New DAT (1979) Effect of oxygen concentration on morphogenesis of cranial neural folds and neural crest in cultured rat embryos. J Embryol Exp Morph 54: 17–35. 528863

[2] Dunwoodie SL (2009) The role of hypoxia in development of the mammalian embryo. Dev Cell 17: 755–773. 20059947 10.1016/j.devcel.2009.11.008

[3] Ke Q, Costa M (2006) Hypoxia-inducible factor-1 (HIF-1). Mol Pharmacol 70: 1469–1480. 16887934 10.1124/mol.106.027029

[4] Tian YM, Yeoh KK, Lee MK, Eriksson T, Kessler BM, et al (2011) Differential sensitivity of hypoxia inducible factor hydroxylation sites to hypoxia and hydroxylase inhibitors. J Biol Chem 286: 13041–1351. 21335549 10.1074/jbc.M110.211110 PMC3075650

[5] Depping R, Steinhoff A, Schindler SG, Friedrich B, Fagerlund R, et al (2008) Nuclear translocation of hypoxia-inducible factors (HIFs): involvement of the classical importin alpha/beta pathway. Biochim Biophys Acta 1783: 394–404. 18187047 10.1016/j.bbamcr.2007.12.006

[6] Fernández-Martínez AB, Jiménez MI, Hernández IS, García-Bermejo ML, Manzano VM, et al (2011) Mutual regulation of hypoxic and retinoic acid related signalling in tubular proximal cells. Int J Biochem Cell Biol. 43: 1198–1207. 10.1016/j.biocel.2011.04.013 21554977

[7] Ito T, Funamoto K, Sato N, Nakamura A, Tanabe K, et al (2012) Maternal undernutrition induces the expression of hypoxia-related genes in the fetal brain. Tohoku J Exp Med 226: 37–44. 22186035 10.1620/tjem.226.37

[8] Keith B, Johnson RS, Simon MC (2011) HIF1α and HIF2α: sibling rivalry in hypoxic tumour growth and progression. Nat Rev Cancer 12: 9–22. 22169972 10.1038/nrc3183 PMC3401912

[9] Lin Q, Cong X, Yun Z (2011) Differential hypoxic regulation of hypoxia-inducible factors 1α and 2α. Mol Cancer Res 9: 757–765. 21571835 10.1158/1541-7786.MCR-11-0053 PMC3117969

[10] Pasanen A, Heikkilä M, Rautavuoma K, Hirsilä M, Kivirikko KI, et al (2010) Hypoxia-inducible factor (HIF)-3alpha is subject to extensive alternative splicing in human tissues and cancer cells and is regulated by HIF-1 but not HIF-2. Int J Biochem Cell Biol 42: 1189–1200. 20416395 10.1016/j.biocel.2010.04.008

[11] Jain S, Maltepe E, Lu MM, Simon C, Bradfield CA (1998) Expression of ARNT, ARNT2, HIF1α, HIF2α and Ah receptor mRNAs in the developing mouse. Mech Dev 73: 117–123. 9545558 10.1016/s0925-4773(98)00038-0

[12] Iyer NV, Kotch LE, Agani F, Leung SW, Laughner E, et al (1998) Cellular and developmental control of O_2_ homeostasis by hypoxia-inducible factor 1 alpha. Genes Dev 12: 149–162. 9436976 10.1101/gad.12.2.149 PMC316445

[13] Ryan HE, Lo J, Johnson RS (1998) HIF-1 alpha is required for solid tumor formation and embryonic vascularization. EMBO J 17: 3005–3015. 9606183 10.1093/emboj/17.11.3005 PMC1170640

[14] Amati F, Diano L, Campagnolo L, Vecchione L, Cipollone D, et al (2010) Hif1α down-regulation is associated with transposition of great arteries in mice treated with a retinoic acid antagonist. BMC Genomics11: 497. 10.1186/1471-2164-11-497 PMC2996993 20846364

[15] Tal R, Shaish A, Barshack I, Polak-Charcon S, Afek A, et al (2010) Effects of hypoxia-inducible factor-1alpha overexpression in pregnant mice: possible implications for preeclampsia and intrauterine growth restriction. Am J Pathol 177: 2950–2962. 20952590 10.2353/ajpath.2010.090800 PMC2993274

[16] Duan LJ, Zhang-Benoit Y, Fong GH (2005) Endothelium-intrinsic requirement for Hif-2alpha during vascular development. Circulation 111: 2227–2232. 15851592 10.1161/01.CIR.0000163580.98098.A3

[17] Scortegagna M, Ding K, Oktay Y, Gaur A, Thurmond F, et al (2003) Multiple organ pathology, metabolic abnormalities and impaired homeostasis of reactive oxygen species in Epas1−/− mice. Nat Genet 35: 331–340. 14608355 10.1038/ng1266

[18] Yamashita T, Ohneda O, Nagano M, Iemitsu M, Makino Y, et al (2008) Abnormal heart development and lung remodeling in mice lacking the hypoxia-inducible factor-related basic helix-loop-helix PAS protein NEPAS. Mol Cell Biol 28: 1285–1297. 18070924 10.1128/MCB.01332-07 PMC2258751

[19] Shum L, Coleman CM, Hatakeyama Y, Tuan RS (2003) Morphogenesis and dysmorphogenesis of the appendicular skeleton. Birth Defects Res C Embryo Today 69: 102–122. 12955856 10.1002/bdrc.10012

[20] Robins JC, Akeno N, Mukherjee A, Dalal RR, Aronow BJ, et al (2005) Hypoxia induces chondrocyte-specific gene expression in mesenchymal cells in association with transcriptional activation of Sox9. Bone 37: 313–322. 16023419 10.1016/j.bone.2005.04.040

[21] Lafont JE, Talma S, Murphy CL (2007) Hypoxia-inducible factor 2alpha is essential for hypoxic induction of the human articular chondrocyte phenotype. Arthritis Rheum 56: 3297–3306. 17907154 10.1002/art.22878

[22] Schipani E, Ryan HE, Didrickson S, Kobayashi T, Knight M, et al (2001) Hypoxia in cartilage: HIF-1alpha is essential for chondrocyte growth arrest and survival. Genes Dev 15: 2865–2876. 11691837 10.1101/gad.934301 PMC312800

[23] Amarilio R, Viukov SV, Sharir A, Eshkar-Oren I, Johnson RS, et al (2007) HIF1alpha regulation of Sox9 is necessary to maintain differentiation of hypoxic prechondrogenic cells during early skeletogenesis. Development 134: 3917–3928. 17913788 10.1242/dev.008441

[24] Webster WS, Abela D (2007) The effect of hypoxia in development. Birth Defects Res C Embryo Today 81: 215–228. 17963271 10.1002/bdrc.20102

[25] Ornoy A, Rand SB, Bischitz N (2010) Hyperglycemia and hypoxia are interrelated in their teratogenic mechanism: studies on cultured rat embryos. Birth Defects Res B Dev Reprod Toxicol 89: 106–115. 20127827 10.1002/bdrb.20230

[26] Huang C, Hales BF (2009) Teratogen Responsive Signaling Pathways in Organogenesis Stage Mouse Limbs. Reprod Toxicol 27: 103–110. 19429390 10.1016/j.reprotox.2009.01.014

[27] Mirkes PE (1985) Cyclophosphamide teratogenesis - A review. Teratogenesis Carcinog Mutagen 5: 75–88. 2859667 10.1002/tcm.1770050202

[28] Hales BF, Jain R (1986) Differential effects of 4-hydroperoxycyclophosphamide on limb development in vitro. Teratology 34: 303–311. 2432673 10.1002/tera.1420340310

[29] Lee YM, Jeong CH, Koo SY, Son MJ, Song HS, et al (2001) Determination of hypoxic region by hypoxia marker in developing mouse embryos in vivo: a possible signal for vessel development. Dev Dyn 220: 175–186. 11169851 10.1002/1097-0177(20010201)220:2<175::AID-DVDY1101>3.0.CO;2-F

[30] Kuschel A, Simon P, Tug S (2012) Functional regulation of HIF-1α under normoxia–is there more than post-translational regulation? J Cell Physiol 227: 514–524. 21503885 10.1002/jcp.22798

[31] Dioum EM, Chen R, Alexander MS, Zhang Q, et al (2009) Regulation of hypoxia-inducible factor 2α signaling by the stress-responsive deacetylase sirtuin 1. Science 324: 1289–1293. 19498162 10.1126/science.1169956

[32] Xu Y, Li Y, Pang Y, Ling M, Shen L, et al (2012) Blockade of p53 by HIF-2α, but not HIF-1α, is involved in arsenite-induced malignant transformation of human bronchial epithelial cells. Arch Toxicol 86: 947–959. 22447124 10.1007/s00204-012-0810-x

[33] Hirao M, Tamai N, Tsumaki N, Yoshikawa H, Myoui A (2006) Oxygen tension regulates chondrocyte differentiation and function during endochondral ossification. J Biol Chem 281: 31079–31092. 16905540 10.1074/jbc.M602296200

[34] Provot S, Zinyk D, Gunes Y, Kathri R, Le Q, et al (2007) Hif-1α regulates differentiation of limb bud mesenchyme and joint development. J Cell Biol 177: 451–464. 17470636 10.1083/jcb.200612023 PMC2064828

[35] Araldi E, Khatri R, Giaccia AJ, Simon MC, Schipani E (2011) Lack of HIF-2α in limb bud mesenchyme causes a modest and transient delay of endochondral bone development. Nat Med 17: 25–26. 21217667 10.1038/nm0111-25 PMC3215585

[36] Huang C, Hales BF (2002) Role of caspases in the murine limb bud cell death induced by 4-hydroperoxycyclophosphamide, an activated analog of cyclophosphamide. Teratology 66: 288–299. 12486762 10.1002/tera.10100

[37] Moallem SA, Hales BF (1998) The role of p53 and cell death by apoptosis and necrosis in 4-hydroperoxycyclophosphamide-induced limb malformations. Development 125: 3225–3234. 9671594 10.1242/dev.125.16.3225

[38] An WG, Kanekal M, Simon MC, Maltepe E, Blagosklonny MV, et al (1998) Stabilization of wild-type p53 by hypoxia-inducible factor 1alpha. Nature. 392: 405–408. 10.1038/32925 9537326

[39] Sermeus A, Michiels C (2011) Reciprocal influence of the p53 and the hypoxic pathways. Cell Death Dis 2: e164. 21614094 10.1038/cddis.2011.48 PMC3122125

